# Ten simple rules for making biomedical data resources accessible

**DOI:** 10.1371/journal.pcbi.1013657

**Published:** 2025-11-06

**Authors:** Thomas C. Smits, Lawrence Weru, Nils Gehlenborg, Sehi L’Yi

**Affiliations:** Department of Biomedical Informatics, Harvard Medical School, Boston, Massachusetts, United States of America; Carnegie Mellon University, UNITED STATES OF AMERICA

## Introduction

Biomedical science relies on data-driven resources that contain highly structured information and many visual components. These biomedical data resources include data repositories and portals, web-based and standalone analysis tools, including visualization tools and computational notebooks. We have performed a large-scale analysis of the state of accessibility of data portals and journal websites, as well as developed various tools to improve the accessibility of biomedical data resources [1–4]. Our 10 simple rules are a combination of lessons we have learned along the way, as well as practical advice for addressing the most common issues we have found.

The accessibility of biomedical data science tools and resources shapes who can participate. Without digital accessibility, biomedical data resources will exclude many of the 1.3 billion people worldwide with disabilities [5]. Accessibility barriers will prevent people with disabilities from entering and working in the biomedical sciences, as highlighted by the significant gap between the number of people with disabilities in the scientific workforce (9%) and the general population (26%) [6,7].

The United Nations describes people with disabilities as “those who have physical, mental, intellectual or sensory impairments, which in interaction with various barriers, may hinder their full and effective participation in society on an equal basis with others” [8]. Various assistive technologies exist to aid in perceiving and operating content, such as screen magnifiers, screen readers, and tactile displays. Here, we highlight how different ways of implementing biomedical data resources impact users with disabilities. We prioritize removing visual and motor barriers, as these are most common in these resources, but the impact extends to others, such as barriers affecting cognition.

According to the United Nations [9], digital accessibility is a fundamental human right. Yet, a recent study of a million websites [10] found an average of 51 accessibility errors per website. As accessibility advocates highlight, organizations often consider digital accessibility a low priority [11], and there is an overall lack of awareness and skills in identifying and addressing accessibility issues [12], which explains the dismal state of accessibility on the web.

We observe the same discrepancy in biomedical science. While there have been efforts to include people with disabilities in the biomedical workforce [13], we have found that almost all biomedical data resources—99.1% of 3,112 data portals and 99.5% of 5,099 journal websites—have critical accessibility issues that are likely to prevent users with disabilities from using them [1]. Improving the digital accessibility of biomedical data resources is not only crucial for helping people with disabilities currently in the workforce, but also critical for opening up biomedical science education and research to future students and researchers with disabilities. Additionally, digital accessibility also benefits the general population, an effect known as the “curb cut effect.” For example, using correct semantic structure in HTML increases reproducibility and understandability of data resources [14].

**Fig 1:**
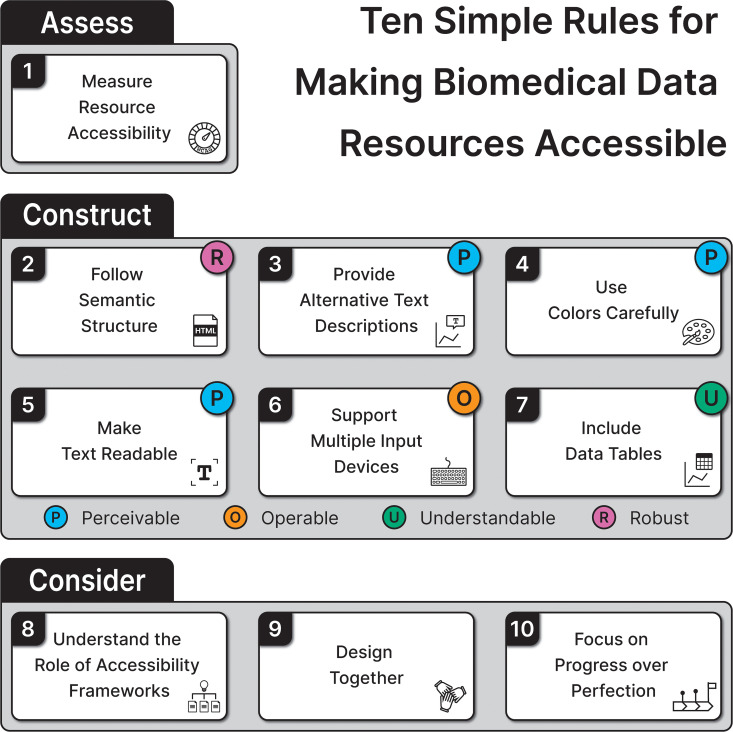
Overview of the 10 simple rules for making biomedical data resources accessible. These rules are grouped into three categories (i.e., assessment, construction, and consideration). The four labels with different colors represent the POUR (Perceivable, Operable, Understandable, and Robust) principles that are related to the corresponding rules.

Our goals for this article are 2-fold. First, we want to raise awareness about the importance of digital accessibility in biomedical research. Second, we offer a set of rules with actionable recommendations for making resources accessible (see Fig 1). Rule 1 covers assessment and lays the groundwork for understanding our focus. Rules 2–7 focus on building accessible resources and provide practical advice on getting started in the field, as well as addressing some of the major accessibility issues frequently found in biomedical data resources. Rules 8–10 cover accessibility standards, frameworks, and broader perspectives. We build upon the extensive guidelines available for digital accessibility, such as the Web Content Accessibility Guidelines (WCAG) [15]. WCAG is organized around four core principles: Perceivable, Operable, Understandable, and Robust (POUR). Our rules follow these principles: Perceivable (Rules 3, 4, 5)—a user must be able to perceive the content (e.g., via alt text for scientific figures to make them accessible for visually impaired users); Operable (Rule 6)—a user must be able to navigate and manipulate the components (e.g., by offering both keyboard and mouse navigation on a websites to make them accessible to users with motor impairments); Understandable (Rule 7)—a user must be able to understand the content and interface (e.g., by using simple language on a website to make it accessible to users who have cognitive impairments); Robust (Rule 2)—content can be interpreted across technologies and platforms (e.g., by designing websites that can be read and controlled with tactile displays in addition to screen readers). These primary categories for individual rules are assigned based on how WCAG and Chartability [16]—accessibility guidelines for visualization—categorize relevant guidelines. Here, we focus on resources built for access through web browsers, although we occasionally highlight their transferability to other mediums (e.g., PDF documents). Our recommendations are meant to highlight the importance of accessibility and provide a starting point for improving accessibility of a resource, but they are not meant to be a comprehensive list for addressing all accessibility barriers. Disabilities are varied, and our suggestions may not impact everyone.

## Rule 1: Measure resource accessibility

Improving accessibility begins with identifying what aspects of a resource are inaccessible.

Accessibility can be evaluated at three levels, each requiring progressively more effort: (1) using automatic accessibility evaluation tools to collect accessibility metrics, (2) manually evaluating with simulated disabilities, and (3) manually evaluating with users with disabilities.

Many tools are available to collect accessibility metrics of resources automatically. Such tools are WebAIM’s Web Accessibility Evaluation Tool (WAVE) [17] and Deque Systems’ Axe Accessibility Testing Engine [18], which identifies accessibility issues in a resource. Such issues are displayed directly on the website, making it easy to identify the elements that need to be fixed. Automatic evaluations provide quick overviews and are suitable for running large-scale analyses of the state of accessibility of resources.

It is essential to note that automatic tools underestimate the actual accessibility issues. Therefore, it is important to perform manual evaluation in addition to using automatic tools. To manually evaluate accessibility for users with visual or motor impairments, a common tool to use is a screen reader. There are several free screen readers, such as VoiceOver on macOS and NVDA on Windows. Screen readers are one of the most commonly used assistive technologies that enable navigation of websites and convert content into speech. Screen readers help determine whether users who rely on them can complete key tasks within a resource. Ultimately, the most accurate way to understand accessibility barriers is to involve users with disabilities in design and testing (see Rule 9).

## Rule 2: Follow semantic structure

All digital accessibility is embedded in structural elements, such as HTML elements for the web. These elements are interpreted by users with or without the aid of assistive technology. Semantic HTML elements are elements with clear functional roles, such as headings and lists. Because semantic elements in HTML are designed and interpreted with specific roles, they bear an innate relation to accessibility [19].

For example, an HTML heading element is automatically styled with a larger font and other markup. This visual style indicates to a seeing user that this is a title. Of course, CSS can be used to create the same appearance on a more general element (e.g., a paragraph), and many users without visual disabilities will not notice the difference. However, using a heading element is key to finding a title for a screen reader user. Therefore, it is important to use heading elements and use them correctly, such as using only one main heading followed by subheadings in logical order without skipping levels, as described in WCAG.

In our large-scale analysis of biomedical data resources, the majority of the top issues were related to incorrect semantic structure [1]. As an illustration, 89% of data portals had incorrect landmark structures, 47% missed main headings, and 42% had unlabeled links.

According to WCAG, a page needs to have landmark elements to separate the header from the main content and the footer. Content types, such as lists, buttons, figures, tables, and captions, should be marked as such, instead of wrapping everything in general content division elements and styling them with CSS. Links need descriptive titles to show what clicking the link leads to.

Following the semantic structure, a low-vision user relying on a screen reader can more easily navigate a data portal to find a data table or a button for downloading the dataset. This furthermore assists users with various cognitive impairments to follow the page structure. This rule also applies to other media, such as markdown, PDF documents, and slide decks.

## Rule 3: Provide alternative text descriptions

Without alternative text (i.e., alt text) for figures, a user who is blind cannot perceive the message that the figure is conveying. Alt text explains what is shown and provides context to a user. Through alt text, the key information presented in a figure can also be perceived by users with low vision.

Alt text should be kept brief, as screen readers do not generally support efficient navigation of alt text. Often, figures can contain complex data visualizations with more information than can be conveyed in a short alt text. W3C guidelines for such figures require a two-part text alternative, i.e., a brief description of the figure and a location for a more detailed description [20]. This way, a user can quickly get an idea of the type of visualization and also reference a longer text describing the visualization in detail.

Lundgard and Satyanarayan [21] introduced a four-level model to understand the content of longer descriptions. They distinguish four categories of information—elemental, statistical, perceptual, and contextual—and highlight that, particularly, the first three categories are regarded as important by users who are blind.

We note that data visualizations can be static or interactive. Most of the existing guidelines focus on static visualizations. Interactive visualizations, such as genome browsers, pose a challenge since text needs to be updated dynamically to reflect the data manipulated in response to user interactions. Moreover, interactive visualizations often support data exploration rather than simply conveying a message, limiting the use of static descriptions. One solution for addressing such challenges is to automatically generate text descriptions based on structured documents used to construct visualizations (e.g., grammar-based specifications or SVG documents). In our work on AltGosling, we employed this approach using a visualization grammar [22] for creating multiple versions of text descriptions for interactive genomics data visualizations [2].

Alt text is not the only text that a figure needs. Figures with data visualizations require descriptive titles, axes, labels, and captions that can help screen reader users understand the data presented in the visualizations and how to interpret them.

## Rule 4: Use colors carefully

Users perceive information through color, so colors need to be used with care in resources. This rule applies to every element that encodes useful information for target users, such as paragraphs on web pages and bars in bar charts.

Most importantly, the color contrast ratio between the foreground and background should be sufficiently high. For example, WCAG 2.2 requires that the ratio is larger than 4.5:1. There are several online tools to calculate the ratio by entering the hex color codes, such as WebAIM’s Contrast Checker [23].

Considering that many users experience color blindness [24], it is desirable to use color palettes that are friendly to users with color blindness. Be cautious when using default color palettes provided by the software, as these are not always accessible. For example, widely-used visualization tools, like IGV [25], often use inaccessible color palettes, so it would be a good practice to change the colors when using them for publication figures (e.g., editing exported vector images). Several accessible palettes are available online, including Okabe and Ito [26] and ColorBrewer [27]. If individual colors are selected, their accessibility needs to be double-checked, for example, using tools that simulate colors for different types of color blindness, such as Colorblindly [28].

Additionally, it’s important not to rely solely on color. If important information is encoded with color in visualizations, it is ideal to redundantly encode such information with other visual channels whenever possible, such as different textures of bars in bar charts or shapes of points in scatter plots. This will not only enable users with low vision or color blindness to gain information from visualizations, but will also be helpful for users without disabilities (e.g., when printed in black and white on paper).

## Rule 5: Make text readable

Making text readable in resources enables low-vision users to perceive important information included in the resources and helps prevent eye strain and fatigue when reading text.

The most commonly suggested guideline is to use fonts no smaller than 12 px (9 pt) [29]. This applies to every text element shown on resources, including not only paragraphs and headings on webpages, but also chart titles and axis titles in visualizations. Only minor elements can be smaller than 12 px, such as axis labels [16]. Common visualization libraries often have too small default font sizes (e.g., 10 px in Matplotlib). Hence, font sizes need to be properly adjusted for accessibility.

In addition, WCAG 2.2 requires that texts are resizable up to at least 200%. This is usually supported by browsers, so no additional implementation is needed in most cases. However, it is still important to test on different browsers.

In addition to font sizes, other factors can influence the accessibility of text. Decorative fonts should not be overly used, such as handwritten fonts. Also, justified text should be avoided, which aligns text to both the left and right sides. The line spacing should be sufficiently large (e.g., at least 1.5 times larger, as specified in WCAG 2.2). Hyperlinks should be visually distinguishable, e.g., by using underlined text and distinct colors. Furthermore, the content should employ clear and straightforward language to support comprehension, particularly for individuals with cognitive disabilities.

## Rule 6: Support multiple input devices

Not everyone uses a mouse or trackpad to navigate biomedical resources. There are other devices available to enable users to navigate interfaces.

Since a majority of assistive devices interact with keyboard APIs, anything that is reachable and operable by a mouse should also be reachable and operable with a keyboard. Interactive components (menus, buttons, filters) need to respond to the Tab and Arrow keys in addition to mouse clicks [30]. When data resources are keyboard operable, they can be used by a broader range of users. For example, supporting keyboard accessibility can reduce mobility and dexterity barriers.

There are many tools available to support multiple input devices. Data Navigator is a framework to support navigation on visualizations with assistive technologies [31]. OpenKeyNav is a JavaScript library that enables keyboard navigation on webpages [4]. Integration of such tools in data portals, such as the Human BioMolecular Atlas Program Data Portal, allows access for users with motor disabilities to use key features that otherwise demand precise mouse movement. This enables users who navigate with keys or use screen readers to reach and navigate data more confidently and independently.

## Rule 7: Include data tables

Figures often rely on data. Even if figures are accompanied by alternative text descriptions, a user who is unable to view the figure may struggle to understand what the data looks like. By including a data table on the webpage near the figure, anyone can view the raw data.

When including a table, its size needs to be carefully determined. Especially in biomedical data resources, data can become very large. A table of 100 rows and 100 columns requires 10,000 keystrokes to view the entire table. Adding a representative subsection of the data (e.g., 5 by 5) or allowing for data filtering helps users digest the information. A study with blind screen reader users found issues with the table size, which had a maximum of 6 columns and 61 rows [32].

This rule also applies to computational notebooks. A comprehensive evaluation [14] revealed that only 4.5% of the notebooks evaluated by the authors had a table near a corresponding figure. For better reproducibility of computational notebooks, the authors argue that raw data should be provided to help other researchers using the notebooks.

## Rule 8: Understand the role of accessibility frameworks

Accessibility advocates have been working to address digital accessibility issues for decades. One of the first mainstream‑computing articles to argue that blind programmers could use computers was published in 1964 [33], and the first version of the WCAG was published in 1999. Individual countries have their own legal frameworks for digital accessibility as well, such as Section 508 in the US, EN 301 549 in the EU, and JIS X 8341-3 in Japan, all of which rely on WCAG. These guidelines and legal framework are publicly available, which can be helpful for learning further about accessibility fundamentals.

There are plenty of resources today for developers of different skill levels to gain proficiency in understanding and implementing accessibility guidelines for different topics. WebAIM [30] and W3Schools [34] provide accessibility training courses and materials. Focusing on the accessibility of biomedical data resources, we organized a tutorial at the ISMB 2025 conference, with tutorial materials publicly available [35]. Different resource types require different accessibility guidelines, and there are many recent technologies in the space of visualizations. For example, Chartability [16] is a set of accessibility guidelines for making interactive data visualization more accessible. To help users with visual impairments understand unfamiliar visualizations, such as Circos [36] and Sequence Logos [37], researchers, including ourselves, explored multiple approaches to effectively describe visual representations in textual descriptions [3,38]. New accessibility-related technologies and tools are continuously published at academic and industry conferences, including ACM SIGACCESS Conference on Computers and Accessibility (ASSETS), ACM CHI Conference on Human Factors in Computing Systems (CHI), IEEE Visualization Conference (VIS), CSUN Assistive Technology Conference, and axe-con Digital Accessibility Conference.

## Rule 9: Design together

Design with people, not for them. When designing solutions for users with disabilities, include them in the design process and get feedback early on. Otherwise, it can cause a so-called “disability dongle” [39], a disability aid that fails to meet the actual needs of users with disabilities due to excluding them during the design process. Although these solutions seem enabling, they actually do not create meaningful access, and instead divert attention away from removing the actual accessibility barriers.

Co-design is a design approach that includes target users in the design process. For accessibility, resources should ideally be co-designed with target users with disabilities [40]. If this is not possible, target users with disabilities need to be included as part of the testing process at the very least. Accessibility issues are easier to fix before deployment. Otherwise, this adds to the tech debt of tools.

There are organizations that can help to find target users with various disabilities to co-design, test, or provide feedback on resource creation. For an academic research project, organizations like the National Federation of the Blind [41] can be contacted to help promote research projects to potential participants. Alternatively, accessibility professionals or organizations can be hired who can test the accessibility of resources, provide feedback on how to address the issues, and also execute the fixes, depending on the scope of work and budget.

## Rule 10: Focus on progress over perfection

We urge resource developers to focus on progress over perfection [42]. 99% of biomedical data resources have “critical” accessibility failures [1]. The accessibility states will remain the same if we wait to fix all issues at once. Accessibility is a spectrum, and addressing a few issues already improves the accessibility of a resource.

The accessibility evaluation results of biomedical data resources [1] can serve as a benchmark for comparing the accessibility of a resource. For example, content creators can focus on fixing the top accessibility issues we identified, make their resources more accessible than the bottom 10% from our results, and then focus on reaching the 25th percentile, progressively increasing accessibility in multiple steps. Each improvement makes resources significantly more accessible to various users.

For certain accessibility barriers, there are no perfect solutions. For example, we explained in Rule 3 how to add alternative text descriptions for visualizations. But, for complex visualizations, there is no single best answer to creating a text description. Any description is better than no description at all. Our manual accessibility evaluation with a blind screen reader user showed that simply following accessibility standards does not guarantee that data portals are accessible [1]. Sometimes creativity is necessary to find solutions to arising accessibility barriers.

## Conclusion

We introduce 10 simple rules for making web-based biomedical data resources accessible and usable. While these are some of the most important guidelines, they do not encompass every aspect of accessibility for all types of biomedical data resources. There are additional accessibility guidelines and tools available to meet the various needs of content creators. Nonetheless, embracing the 10 simple rules presented here will lead to a better understanding of the resources and tools available and serve as a foundation for further learning. The tools mentioned in each rule are summarized in S1 Table (see Supporting information).

Better accessibility improves usability for everyone. Whether aging, recovering from an injury, juggling tasks in a busy environment, caring for someone with access needs, or living with a disability, accessibility will eventually become relevant to all of us, if it is not already. In particular, accessibility is relevant for education and research in computational biology. As computational biologists, we share a mission to make our data and tools usable and accessible to everyone [43]. Making our resources more accessible will increase the impact of our research on the community.

We invite the computational biology community to join us in taking action. Although WCAG has been around for over 25 years, the vast majority of resources remain inaccessible [1]. As we emphasized earlier, every small change that each of us makes can lead to a significant impact on the community. Only together can we make our field more open and accessible.

## Supporting information

S1 Table The summary of accessibility tools mentioned in our 10 simple rules. Their web pages are assessed on October 7th, 2025.
(PDF)
