# Supplementary material for: Ten simple rules for making biomedical data resources accessible
Source: PLoS Comput Biol. 2025 Nov 6;21(11):e1013657. doi: 10.1371/journal.pcbi.1013657 (PMC12591410; doi:10.1371/journal.pcbi.1013657)
Supplement: S1 Table — Their web pages are assessed on October 7th, 2025. (PDF) [file pcbi.1013657.s001.pdf]

Supplemental Materials

Table S1. The summary of accessibility tools mentioned in our Ten Simple Rules. Their web pages are assessed on October 7th, 2025.

Resource	Link	Section
WAVE Web Accessibility Evaluation Tool	https://wave.webaim.org/	Rule 1: Measure Resource Accessibility
Axe Accessibility Test Engine	https://www.deque.com/axe/	Rule 1: Measure Resource Accessibility
VoiceOver	https://support.apple.com/guide/voiceover/welcome/mac	Rule 1: Measure Resource Accessibility
NVDA	https://www.nvaccess.org/download/	Rule 1: Measure Resource Accessibility
AltGosling	https://gosling-lang.github.io/altgosling/docs/	Rule 3: Provide Alternative Text Descriptions
WebAIM Contrast Checker	https://webaim.org/resources/contrastchecker/	Rule 4: Use Colors Carefully
Okabe and Ito	https://siegal.bio.nyu.edu/color-palette/	Rule 4: Use Colors Carefully
ColorBrewer	https://colorbrewer2.org/	Rule 4: Use Colors Carefully
Colorblindly	https://github.com/oftheheadland/Colorblindly	Rule 4: Use Colors Carefully

Data Navigator	https://www.frank.computer/data-navigator/	Rule 6: Support Multiple Input Devices
OpenKeyNav	https://openkeynav.com/	Rule 6: Support Multiple Input Devices
Chartability	https://chartability.fizz.studio/	Rule 8: Understand the Role of Accessibility Frameworks
